# Are There Effective Intervention Measures in Broiler Production against the ESBL/AmpC Producer *Escherichia coli*?

**DOI:** 10.3390/pathogens10050608

**Published:** 2021-05-15

**Authors:** Evelyne Becker, Michaela Projahn, Elke Burow, Annemarie Käsbohrer

**Affiliations:** 1 Department for Biological Safety, German Federal Institute for Risk Assessment, 12277 Berlin, Germany; michaela.projahn@bfr.bund.de (M.P.); elke.burow@bfr.bund.de (E.B.); annemarie.kaesbohrer@bfr.bund.de (A.K.); 2 Institute of Pharmacy/LPG, Pharmaceutical Biology, Universität Greifswald, 17489 Greifswald, Germany; 3 Unit of Veterinary Public Health and Epidemiology, University of Veterinary Medicine Vienna, 1210 Wien, Austria

**Keywords:** broiler, poultry, antibiotic resistance, ESBL, AmpC, *E. coli*, one health, food chain, biosecurity, control measure, intervention measure

## Abstract

Extended-spectrum beta-lactamase (ESBL) and AmpC beta-lactamase (AmpC) producing Enterobacteriaceae occur frequently in livestock animals and the subsequent stages of the meat production chain and are therefore considered a risk for human health. Strict biosecurity measures and optimal farm management should reduce or even prevent poultry flock colonization at farm level. This review summarizes and evaluates published information on the effectiveness of specific intervention measures and farm management factors aiming to reduce the occurrence and spread of ESBL/AmpC producing or commensal or pathogenic *E. coli* in broiler chicken farms. In this systematic literature review, a total of 643 publications were analyzed, and 14 studies with significant outcome about the effectiveness of specific measures against *E. coli* and ESBL/AmpC producing *E. coli* in broiler chicken farms were found. Different feed additives seem to have an impact on the occurrence of those microorganisms. The measures ‘cleaning and disinfection’ and ‘competitive exclusion’ showed strong effects in prevention in some studies. In summary, some intervention measures showed potential to protect against or eliminate ESBL/AmpC-producing, commensal or pathogenic *E. coli* at farm level. Due to the high variability in the outcome of the studies, more specific, detailed investigations are needed to assess the potential of the individual intervention measures.

## 1. Introduction

The World Health Organization (WHO) has declared antibiotic resistance to be one of the biggest threats to global health, food-security, and development [1]. One of the most widespread resistance mechanisms of bacteria is the enzymatic inactivation of antibiotics by beta-lactamases. In that process, the beta-lactam ring of the antibiotics is hydrolyzed, and thus the irreversible blockade of the enzyme essential for the cell wall synthesis of these bacteria is prevented. The occurrence of ESBL-producers and AmpC-producers in Enterobacteriaceae like *Escherichia coli* has increased. They were frequently detected in livestock, companion, and wildlife animals [2,3], in humans, vegetables and broilers [4,5] but also in water, soil, air, or dust [6,7,8]. In Germany, high detection rates for ESBL/AmpC producing *E. coli* were identified in broiler houses and broilers [9,10,11]. The possible transmission to humans may occur via direct contact with animals or the environment, or via the consumption of contaminated food [5,12,13].

All levels of the broiler production chain have been investigated: the hatchery [14,15], the broiler farm [9,10,11], the slaughterhouse [16,17], and the fresh meat [18,19]. The highest prevalence was found in the broiler chicken fattening farms [9,20]. Furthermore, studies on vertical and horizontal transmissions [11,15,20] as well as transmission dynamics [5] have been performed.

So-called biosecurity measures shall prevent potentially pathogenic microorganisms from entering a broiler farm and spreading within the farm. External measures shall protect the animals from pathogens in the environment, that enter the farm by vectors such as transport vehicles [21], humans [22,23,24], companion animals [25,26,27], wild animals [28,29,30], rodents [31,32], and water or feed [20,33,34,35,36]. Internal biosecurity aims to prevent the spread of pathogens within the farm, for example through hygiene measures such as protective clothing, hand washing or cleaning, and disinfection of the pens [37,38,39]. Various management factors can also have an influence on the occurrence of diseases: ventilation and temperature, litter quality, stocking density, breed, and housing conditions or an all-in-all-out-system [40,41,42]. Finally, measures are discussed, which can improve animal health by helping to strengthen the immune system or prevent colonization of the gut by pathogenic bacteria [43,44,45].

Several intervention measures have been successfully applied to control *Salmonella* in livestock animals [46,47] but they were identified not to be effective against *Campylobacter* or ESBL/AmpC-producers, as demonstrated by the wide spread and increasing numbers of these microorganisms [48,49].

Therefore, this review aims at evaluating the effectiveness of measures to reduce commensal or pathogenic *E. coli* as well as ESBL/AmpC producing *E. coli* on broiler farms.

## 2. Results

### 2.1. Literature Search

**Figure 1:**
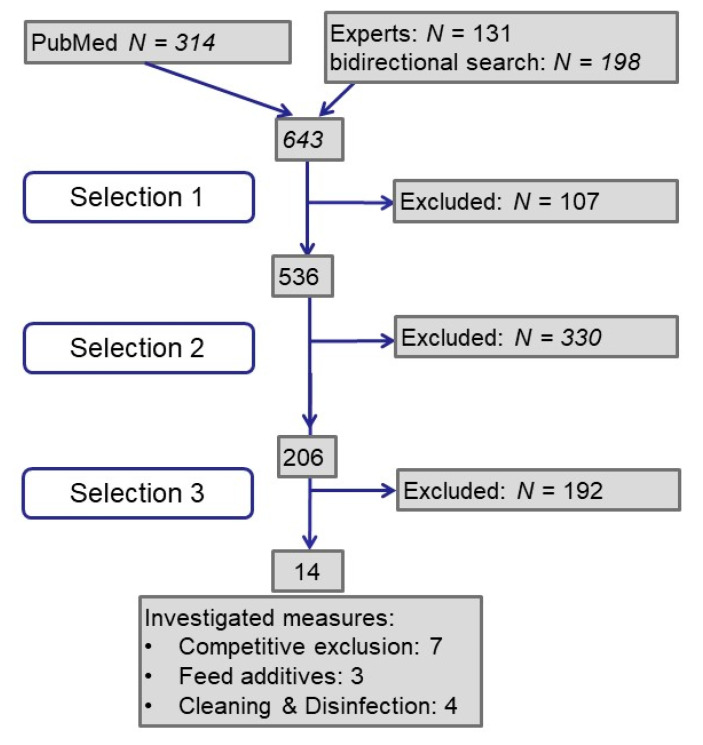
Flow diagram on process of literature retrieval and selection.

A total of 643 publications were analyzed (see Figure 1): Half of them resulted from the PubMed search by keywords, one quarter each from experts and by bidirectional literature search. In the first selection step we excluded publications for not meeting our requirements concerning language and peer reviewed publication of primary data. In the next step, we searched for studies investigating ESBL/AmpC-producing *Escherichia coli* in poultry or broiler or chicken or layer but did not exclude studies about commensal or pathogenic *E. coli*. With the third step we selected studies investigating the effect of intervention measures in broiler houses or broilers on farm level. Finally, we identified 14 publications.

These 14 articles reported results from eight different countries: the seven European countries, Austria, Belgium, Denmark, Finland, Germany, Netherlands, and Poland (13 publications) and one non-European country: China (1).

### 2.2. Intervention Measures

The articles identified from the literature search investigated the intervention measures cleaning and disinfection (4), competitive exclusion (7) and feed additives (3). The respective results are summarized in Table 1.

#### 2.2.1. Competitive Exclusion

Half of the identified publications (7/14) studied the effect of a probiotic-treatment on the presence of microorganisms in the gastrointestinal tract of broilers and their excretion. Almost all of them (6/7) investigated ESBL [52,55,56], or ESBL and AmpC producing *E. coli* [51,53,54], one study focused on the poultry pathogenic *E. coli* O20:K-:H8 and the human pathogenic *E. coli* O157:H7 [50].

The commercial product Aviguard (MSD Animal Health Nederland, Boxmeer, the Netherlands) was investigated by five of the studies [52,53,54,55,56], two studied the product Broilact (Orion Corporation, Espoo, Finland) [50,51]. These competitive exclusion products shall reflect the intestinal microflora of poultry. Dame-Korevaar et al. [55] additionally studied the effect of the synbiotic product PolyStar (Biomin Holding GmbH, Getzersdorf, Austria), consisting of probiotic microorganisms and prebiotic fructooligosaccharides.

Ceccarelli et al. [52] tested in a seeder-bird model the impact of treating one-day-old broilers with the commercial product Aviguard before challenging the seeder birds the next day with 10^6^ CFU/mL *E. coli* strain E75.01/pE38.27 (ESBL). The average excretion until day 14 for the control group (no Aviguard) was 5.68 log_10_ CFU/g feces compared with 1.17 log_10_ CFU/g feces in the group where challenged and not challenged chicks received CE flora. They measured 2.22 log_10_ CFU/g feces in the group in which only challenged chicks received CE flora and 3.86 log_10_ CFU/g feces in the group in which only non-challenged chicks received CE flora, respectively. The resulting differences are 4.5 log_10_, 3.46 log_10_, and 1.82 log_10_ CFU/g feces. Methner et al. [53] and Methner, Rösler [54] investigated the effect of Aviguard applied on day 1 of life to the colonization of seven or four ESBL- or AmpC-producing *E. coli* strains administered the next day in different doses (2 × 10^4^ to 2 × 10^8^ CFU/bird) in layer birds or broilers. The effect differs for the strains and breeds but can achieve a reduction of 4–5 log_10_ CFU/g in layer birds, when exposed to seeder birds (1:5) infected with 1–2 × 10^5^ CFU/bird. With Ross 308 broilers a reduction of 2.5–3.5 log_10_ CFU/g was achieved in this seeder-bird-experiment. Even when challenging the birds with high doses of ESBL- or AmpC-producing *E. coli* strains (10^6^ to 10^8^ CFU/bird), a reduction of about 2 log_10_ CFU/g was found in cecal content. Dame-Korevaar et al. published in 2020 [55,56] results of several seeder-bird experiments with prolonged supply of CE-cultures from day 0 until day 7 or even day 14 of life, twice a day. First [55], they investigated time until colonization, excretion and transmission for a CTX-M-1 *E. coli* (ESBL-producer) and application of the CE-products Aviguard or PoultryStar for fourteen days. Both products delayed the time until colonization (hazard ratio between 3.71 × 10^−3^ and 3.11) in all birds, when the seeder-birds (1:1 seeder:contact) were challenged on day 5 with 10^2^ CFU/mL, reduced the excretion of CTX-M-1 *E. coli* and the excretion of total *E. coli* (up to −1.60 log_10_ CFU/g). The concentration in cecal content was slightly lower (up to −2.80 log_10_ CFU/g) and a 1.5 to 3-fold reduction in transmission rate was observed. Later in 2020 Dame-Korevaar et al. [56] published experiments where they administered the CE-product Aviguard for seven days and challenged the seeder-birds on day 5 (seeder:contact 1:5) with 10^5^ CFU/mL CTX-M-1 *E. coli* and none of the broilers were positive for the challenging ESBL-producing strain. In the control group, 93.5% of the birds were colonized. They also observed that contact birds were colonized later than the seeder-broilers (Time to event Ratio 3.53, 95% CI 3.14 to 3.93) and the microbiota composition was more diverse in CE-broilers than in control broilers at days 5 and 21.

For Broilact a minimal reduction of 2.0 and a maximum reduction of 5.5 log_10_ CFU/g cecal contents of *E. coli* CK11ctx (ESBL producer) was reported by Nuotio et al. [51]. Moreover, a minimum reduction of 2.2 and a maximum reduction of 5.5 log_10_ CFU/g in cecal contents of *E. coli* O20:K-:H8 was found by Hakkinen et al. [50]. They reported a minimum reduction of 0.9 and a maximum reduction of 6.3 log_10_ CFU/g cecal contents for another strain, *E. coli* O157:H7, in broilers. In both studies, the probiotic product was applied to newly hatched broilers and the challenge was performed the next day. The application of Broilact was slightly different, because Hakkinen et al. administered 1 mg of Broilact in 0.5 mL dechlorinated water, while Nuotio et al. used 1 mg Broilact in 0.3 mL. The challenge dose was also different: Hakkinen et al. used 10^3^ viable *E. coli* O20:K-:H8 organisms per bird or 10^5^ *E. coli* O157:H7, whereas Nuotio et al. used a dilution of 10^4^ of *E. coli* strain CK11ctx.

#### 2.2.2. Cleaning and Disinfection

In our literature search, four of the 14 relevant publications (28.6%) studied the effect of cleaning and disinfection in livestock houses. Luyckx et al. [57] evaluated four different cleaning protocols in broiler houses. They included dry cleaning, wet cleaning, and disinfection. Differences concerned, for example, the duration of the steps and the temperature of the water. From the swab samples taken before cleaning 97% were positive for *E. coli*, but after disinfection, only 7% were positive, which means 86% of the *E. coli* were eliminated by cleaning and disinfection. No differences were found between the different cleaning and disinfection protocols. A second study of Luyckx et al. [58] investigated different sampling methods and the significance of different microorganisms as hygiene indicators. They found a total reduction of *E. coli* by 1.6 log_10_ CFU/625 cm^2^ from swab samples in broiler houses by cleaning and disinfection. Cleaning, had a decreasing effect of 1.3 log_10_ CFU/625 cm^2^, whereas disinfection resulted in a reduction of 0.3 log_10_ CFU/625 cm^2^. Gradel et al. [59] investigated a temperature–humidity–time treatment in layer houses by using steam treatment with or without 30 ppm formaldehyde. The most effective method was the application of steam-treatment with 30 ppm formaldehyde for 24 h and it resulted in 100% elimination of naturally occurring *E. coli* in feces samples. The fourth article is about the use of slightly acidic electrolyzed water (SAEW) as an alternative disinfectant in layer houses, investigated by Hao et al. [60]. When the SAEW was sprayed with a high-pressure sprayer at a rate of 120 mL/m^2^ for 5 min in layer houses, the isolation rates of *E. coli* showed a decrease of 16% compared to samples taken before disinfection.

#### 2.2.3. Feed Additives

The impact of feed additives on the gut microbiota was tested in three studies. The microorganisms analyzed were ESBL/AmpC producing *E. coli* (1 publication) and *E. coli* (2), the livestock animals investigated were broilers in all cases.

Jamroz et al. [62] achieved a reduction of *E. coli* by 0.84 log_10_ CFU/g intestinal digesta with a combination of carvacrol, cinnamaldehyde and capsaicin in a diet based on maize and a reduction of even 1.6 log_10_ CFU/g intestinal digesta with this combination of plant extracts in a diet based on wheat and barley.

Goodarzi Boroojeni et al. [61] investigated the thermal processing of feed and the inclusion of organic acids in broiler diets. They supplemented the feed with three different levels of a commercial product containing formic acid and propionic acid. Furthermore, it was treated with four different types of thermal processes. Neither for the thermal treatment nor for the organic acid supplementation significant results were observed. However, the measured cell number of *E. coli*/Hafina/Shigella was the lowest for treatment of the feed with 0.75% acid in the crop, in the ileum and in the caecum.

Roth et al. [63] investigated in their study the effect of a commercial product containing organic acids (formic, acetic, and propionic acids) and, additionally, essential oils (cinnamaldehyde) on the prevalence of resistant *E. coli*. This feed additive based on formic (20%) and acetic (10%) and propionic acids (5%) as well as 2.5% cinnamaldehyde, was applied at a dosage of 2 kg/t of feed for 38 days to 480 broiler chickens. This product showed a about 1.84 log_10_ CFU/g lower count of ESBL-*E. coli* in broiler cecum on day 38 in the group receiving feed-additives compared to the control group, but this effect was not significant.

## 3. Discussion

The occurrence of ESBL/AmpC producing *E. coli* in broiler meat is possessed as a risk for human health, as the transmission to humans is largely confirmed [64,65] and may lead to difficulties in the treatment of diseases [66]. To combat this danger, the broiler production chain is under observation and intervention measures are investigated to prevent the entry into the animals and the spread beneath the animals, the flocks, the pen, or even the environment.

In this review, we summarize the results from intervention studies aiming at reducing *E. coli*, commensal, pathogenic and ESBL-/AmpC-producing, in broiler and broiler chicken farms.

We used different approaches and search strategies and identified 14 articles (2.2%) as matches for our research question. This yield is quite in line with other systematic reviews [66,67,68]. Nevertheless, we have tried to find the balance between specific and general search parameters. For example, we have been simultaneously searching for all intervention methods with general key words like “risk” or “effect”, instead of searching all known intervention measures like “disinfection” or “feed additives separately. This led to a greater yield but also to unspecific results. However, it allowed us to find measures that were unknown to us. Furthermore, we did not restrict the literature search to title and abstract, which unfortunately also led to results in the references of the publications. We therefore decided to complement the database search with the references and studies citing relevant articles, starting with already identified publications. This approach, the so-called bidirectional search, promises numerous results in finding relevant literature [69]. Nevertheless, 14 articles is a small number and only three intervention measures have been investigated. The effectiveness of control measures against ESBL-AmpC-producing *E. coli* in broiler fattening farms seems to be an underreported topic. However, we expected the intervention measures to aim on combating these microorganisms in the broiler farm like cleaning and disinfection or in the broiler itself, as competitive exclusion and feed additives do. The largest number of publications were on competitive exclusion (50%), a method known since 1973, were Nurmi and Rantala [70] proved the effectiveness for *Salmonella*. In the last decades it has been further developed and different microorganisms and commercial products have been tested [50,71,72]. The principle is as complex as the microbiota of broilers. Nevertheless, it seems, as if there is a significant effect, when applying CE-cultures preventively [50,51,52,53,54,55,56]. Although various animal trials were conducted with various CE-products and challenging strains of *E. coli*, they all discovered a maximum reduction of around 5 log_10_ CFU/g cecal content or even complete prevention of colonization. Competitive exclusion seems to be a potential intervention method to suppress colonization of pathogenic or ESBL- or AmpC-producing *E. coli*, as applied, in broiler chickens if used very early in life and depending on the strains and amounts used for protection and challenge.

Cleaning and disinfection is the standard approach to eliminating pathogens from hands, boots, equipment, transport vehicles and the stables between the production cycles. It has been known to be an important part of the reduction strategy against pathogenic bacteria for a long time [73]. Unfortunately, the cleaning protocols and the detergents and disinfection substances are variable and lead to various results, possibly, to transmission to subsequent flocks, as Daehre et al. have proven recently [11]. Therefore, the general effect for cleaning and disinfection differed from small (16% and 1.6 log_10_ reduction) to big (86% or even 100% elimination). In addition to the cleaning and disinfection substance, the number of substances used and the method as well as the temperature of the water, further factors might have an impact on the effectiveness as exposure time or handling differences due to different persons involved [39,74,75]. Not to forget that the long-term use of disinfectants can have opposite effects, such as the development of cross-resistances or the increased formation of biofilm [76,77].

Feed additives like essential oils, organic acids but also enzymes or phytochemicals can improve feed intake or nutrient digestibility [78,79,80,81]. Due to the variety of the active ingredients, the same great variability in the effect is not surprising. However, if one considers the results of the studies found for this review, their efficacy seems to be rather small, regardless of whether the essential oils and organic acids were applied alone or in combination [61,62,63]. In contrast, a more recent study found a synergistic effect for organic acids and essential oils in the reduction of *E. coli* in broilers [82]. It seems as if feed additives provide again a broad range of substances and application methods and therefore their effectiveness for reducing ESBL/AmpC-producers in the broiler production is still under investigation.

In summary, we have found few and very different data for the effectiveness of intervention measures against ESBL/AmpC-producing, commensal or pathogenic *E. coli* in the broiler production. On the one hand other authors concluded that there is no such thing as a totally biosecure farm [83], and there is no route, vehicle, or vector which can be clearly identified as a unique target for intervention [84]. Moreover, Dame-Korevaar et al. [49] found limited to no causal evidence of transmission along the transmission routes investigated. On the other hand, the issue of biosecurity is omnipresent [85]. In this respect, the validation of biosecurity protocols needs to be strengthened, otherwise the farmer may not be using time and money effectively [86]. Fortunately, some of the intervention measures can have a great impact, as can be seen from the results presented. Additional publications present interesting results for other livestock animals, microorganisms for intervention measures such as a prolonged vacancy period [87], different litter materials [88,89] or acidified litter [90]. The results are also variable, ranging from no effect [87,89] to a reduction of 5 log_10_ CFU/g [88]. These studies should be carried out for poultry and ESBL/AmpC-producing *E. coli* as well. Thus, further and more detailed studies are needed to investigate these practices and other risk factors.

## 4. Materials and Methods

### 4.1. Literature Search

A systematic literature review was conducted. The literature search was performed by using online databases and additional bidirectional searching for references and publications citing relevant articles. First, we searched the online database PubMed using terms reflecting possible risk factors like biosafety, internal and external biosecurity, and management factors with impact on the occurrence of pathogens as well as non-pathogenic microorganisms in livestock farming (Table 2). The transmission routes into a farm and from a farm into a slaughterhouse were considered as well. Therefore, these keywords were included. The starting point for the bidirectional search (bidirectional citation searching to completion, BCSC-Method; [69] were articles already identified by experts as potentially relevant (project proposal of EsRAM (German acronym for “Development of interventions to reduce antimicrobial resistance in pathogens in the broiler production”)). Their references and the publications citing them were investigated.

### 4.2. Data Selection

Publications in international journals were included in the evaluation. We did not exclude any study due to its origin country or publication date.

#### Inclusion or Exclusion of Publications

Within the first selection step we excluded articles in other language than English or German and selected publications with primary data, peer-reviewed from international journals. In the next step these studies were analyzed for the livestock animal and the investigated microorganism. The animal production groups broiler, layer, chicken, turkey and duck, poultry in general, and the microorganisms ESBL/AmpC producing *E. coli* but additionally commensal/non-resistant *E. coli* in general were included. All other sources of samples, e.g., cattle, pigs, humans, rodents, environment or meat, and investigated bacteria (Enterobacteriaceae, *Campylobacter*, *Salmonella*) but also viruses and combinations like total mesophilic aerobic bacteria as well as other resistances were excluded. In the third step the articles were analyzed by the aim of the study. Only articles about specific measures to reduce bacterial occurrence in livestock animals or livestock farms were kept.

### 4.3. Calculating the Effectiveness

The published results for the reduction of relevant microorganisms were converted, if necessary, into a log_10_-reduction. For example: 2.04 × 10^7^ = log_10_ (20400000) = 7.3 log_10_.

### 4.4. Definition of Intervention Measures

General biosecurity measures are expected to prevent potentially pathogenic microorganisms entering a broiler farm and spreading within the farm. Those measures are divided into external and internal biosecurity measures. External actions are supposed to protect the animals from pathogens in the environment, entering the farm by vectors like transport vehicles, farm workers, visitors, equipment, feed, water, rodents, or pests. The internal biosecurity should prevent the spread of pathogens within the farm [91,92]. These extensive biosecurity methods and recommendations are complex and their effectiveness on the whole is hard to evaluate.

For our review we looked for focused intervention methods which were applied once or at least for a short time to the chickens or the chickens houses/pens and are expected to lead to measurable effects on the prevalence of ESBL/AmpC producing *E. coli* or approximately *E. coli* as closest relatives within the species.

## Figures and Tables

**Table 1:** Articles about the effectiveness of intervention measures against ESBL/AmpC-resistant *E. coli* or *E. coli* (n = 14).

Articles	Animal/ Breed	Matrix	Microorganism/Strain	Relevant Substance	Min Reduction	Max Reduction
Intervention measure competitive exclusion						
Hakkinen, Schneitz, 1996 [50]	broiler (Ross 1)	cecal content	*E. coli* (O20:K-:H8) *E. coli* (O157:H7)	commercial product Broilact (Orion Corporation, Espoo, Finland)	0.9 log_10_ CFU/g (*E. coli* O157:H7) 2.2 log_10_ CFU/g (*E. coli* O20:K-:H8)	6.6 log_10_ CFU/g (*E. coli* O157:H7) 5.5 log_10_ CFU/g (*E. coli* O20:K-:H8)
Nuotio et al., 2013 [51]	broiler (Ross 508)	cecal content	ESBL-prod. *E. coli* (CK11ctx) AmpC-prod. *E. coli* (CK23ctx; CK68ctx)	CE: commercial product Broilact	2 log_10_ CFU/g (CK11ctx) 2 log_10_ CFU/g (CK23ctx) 1 log_10_ CFU/g (CK68ctx)	5.5 log_10_ CFU/g (CK11ctx) 4 log_10_ CFU/g (CK23ctx) 4 log_10_ CFU/g (CK68ctx) **
Ceccarelli et al., 2017 [52]	broiler	feces	ESBL-prod. *E. coli* (E75.01/pE38.27)	commercial product Aviguard (MSD Animal Health Nederland, Boxmeer, the Netherlands)	1.82 log_10_ CFU/g	4.5 log_10_ CFU/g
Methner et al. 2019 [53]	layer (White Leghorn)	cecal content	ESBL-prod. *E. coli:* EEC 1475N - blaCTX-M15; EEC 1476N - blaTEM-52; EEC 1477N - blaTEM-20; EEC 1478N - blaSHV-12; EEC1500N - blaSHV-12/TEM; EEC1501N - blaCTX-M1); AmpC-prod. *E.* *coli:* (EEC 1479 N-blaCMY-2)	commercial product Aviguard	2.0 log_10_ CFU/g	ca. 4.0–5.0 log_10_ CFU/g (strain variations)
Methner, Rösler, 2020 [54],	layer (White Leghorn (WL)) broiler (Ross 308)	cecal content	ESBL-prod. *E. coli:* (EEC 1475N - blaCTX-M15; EEC 1476N - blaTEM-52; EEC 1478N - blaSHV-12); AmpC-prod. *E. coli:* (EEC 1479 N-blaCMY-2)	commercial product Aviguard	WL: 2.5–3.0 log_10_ CFU/g Ross: 2.5–3.5 log_10_ CFU/g	WL: 5.0–6.0 log_10_ CFU/g Ross: 3.0–3.5 log_10_ CFU/g
Dame-Korevaar et al., 2020 [55]	broiler (Ross 308)	feces	total *E. coli* and ESBL-prod. *E. coli* (strain E38.27)	commercial product Aviguard or PoultryStarsol (Biomin Holding GmbH, Getzersdorf, Austria; SYN)	CEP: no difference in the hazard ratio but reduction of total *E. coli* concentrations (−0.36, 95% CI −0.63 to −0.08 log_10_ CFU/g cecal content).	CEP or SYN: partially prevention of colonization, reduced time until colonization (hazard ratio between 3.71 × 10^−3^ and 3.11), reduced excretion (up to −1.50 log_10_ CFU/g), reduced cecal content (up to −2.80 log_10_ CFU/g), a 1.5 to 3-fold reduction in transmission rate.
Dame-Korevaar et al., 2020 [56]	broiler (Ross 308)	feces and cecal content	ESBL-prod. *E. coli* (strain E38.27)	commercial product Aviguard	Delayed time until colonization: Time Ratio (TR) 3.00, 95% CI 1.82 to 4.95, TR 3.53, 95% CI 3.14 to 3.93.	broilers in the CE groups were not colonized
Intervention measure cleaning and disinfection						
Luyckx et al., 2015 [57]	broiler	surface	*E. coli*	cleaning: commercial solutions containing sodium hydroxide disinfection: a combination of quaternary ammonium compounds (quats), aldehydes and alcohol	na	86% reduction in number of positive swab samples only little differences (1–3%) for the options soaking step and using warm or cold water for cleaning
Luyckx et al., 2015 [58]	broiler	surface	*E. coli*	cleaning compounds: Sodium hydroxide disinfection compounds: Quaternary ammonium compounds, aldehydes, alcohols	na	cleaning: 1.3 log_10_ CFU/625 cm^2^ disinfection: 0.3 log_10_ CFU/625 cm^2^
Gradel et al., 2004 [59]	layer	feces/feed	E. coli *	humidity, formaldehyde	na	100% elimination of naturally occurring *E. coli* in feces samples
Hao et al., 2013 [60]	layer	surfaces, feces, feed, feathers and dust	*E. coli*	slightly acidic electrolyzed water (SAEW, pH 5.0–6.5) with an available chlorine concentration of 300 mg/L	na	16% reduction in number of *E. coli* positive samples
Intervention measure feed additives						
Goodarzi Boroojeni et al., 2014 [61]	broiler (Cobb)	digesta from crop, gizzard, cecum and ileum	*E. coli*	commercial product containing 63.75% formic acid, 25.00% propionic acid and 11.25% water	1.5% acid: 0.6 log_10_ CFU/g (not significant)	0.75% acid: 0.7 log_10_ CFU/g (not significant)
Jamroz et al., 2005 [62]	broiler (Hubbard Hi-Y)	contents of the small intestine, whole caeca	*E. coli*	commercial product containing carvacrol 49.5 g/kg, cinnamaldehyde 29.7 g/kg and capsaicin 19.8 g/kg	treatment-diet based on maize: 0.84 log_10_ CFU/g intestinal digesta	treatment-diet based on wheat and barley: 1.6 log_10_ CFU/g intestinal digesta
Roth et al., 2017 [63]	broiler (Ross 308)	cecal content	ESBL-prod. *E. coli*	commercial product containing 20% formic, 10% acetic, 5% propionic acids, and 2.5% cinnamaldehyde	no effect	1.84 log_10_ CFU/g (not significant)

ESBL-prod. *E. coli* = ESBL-producing *E. coli*, AmpC-prod. *E. coli* = AmpC-producing *E. coli*; * *E. coli* were organic indicator samples as it was too hazardous to put *Salmonella* samples into the layer houses. ** Estimated numbers from box-plots: Effect on *E. coli* CK11ctx (ESBL): reduction from (3.5, 4, 5, 6) to (1, 0.5, 3, 0.5) ≥ min 2, max 5.5 log_10_ Effect on *E. coli* CK23ctx: reduction from (5, 5, 5, 5) to (7, 7, 9, 9) ≥ min 2, max 4 log_10_ Effect on *E. coli* CK68ctx: reduction from (4, 6, 3.8, 6) to (3, 3.8, 0, 2) ≥ min 1, max 4 log_10_.

**Table 2:** Search terms used in the database PubMed, search performed in January 2018 and updated in February 2021.

Search	Operator	Search Term	Purpose
#1		broiler * OR chick * OR poultry	Search for broiler or close relatives
#2	AND	farm OR hatch * OR slaughterhouse	Search for broiler production sites
#3	AND	risk * OR prevention OR management OR control OR intervention OR measure OR effect	Search for intervention measures
#4	AND	enterobacter * OR *escherichia* OR “ESBL” OR “*E. coli*”	Search for ESBL and close relatives
#5	AND	((antimicrobi * AND resistan *) OR (antibiotic * AND resistan *))	Search for antimicrobial resistance in the micro-organisms

asterisk (*) is a wildcard character to include alternative word forms, plurals etc.
